# Enhanced Model Predictive Control Using State Variable Feedback for Steady-State Error Cancellation

**DOI:** 10.3390/s24185869

**Published:** 2024-09-10

**Authors:** Marcos Andreu, Jaime Rohten, José Espinoza, José Silva, Esteban Pulido, Lesyani Leon

**Affiliations:** 1 Department of Mining and Geological Engineering, The University of Arizona, Tucson, AZ 85719, USA; mandreu@arizona.edu; 2 Department of Electrical and Electronic Engineering, Universidad del Bío-Bío, Concepción 4051381, Chile; jsilva@ubiobio.cl (J.S.); lleon@ubiobio.cl (L.L.); 3 Department of Electrical Engineering, Universidad de Concepción, Concepción 4070386, Chile; jose.espinoza@udec.cl; 4 Department of Electrical Engineering, Universidad Técnica Federico Santa María, Valparaíso 2390123, Chile; esteban.pulido@usm.cl

**Keywords:** predictive control, voltage-source converters, optimal control, steady-state error

## Abstract

The rapid dynamic responses of predictive control algorithms are widely acknowledged. However, achieving accurate steady-state reference tracking hinges not just on a precise mathematical model of the system but also on its parameters. This document presents a predictive control scheme augmented with integral state feedback tailored to a photovoltaic (PV) application. In scenarios with uncertain system parameters, steady-state errors can particularly impact reactive power regulation, where the absence of an integral term in the loop exacerbates this issue. The robustness and sensitivity of both predictive control and the proposed enhanced predictive controller are thoroughly examined. Simulation and experimental results are included to validate the effectiveness of this approach.

## 1. Introduction

**Figure 1:**
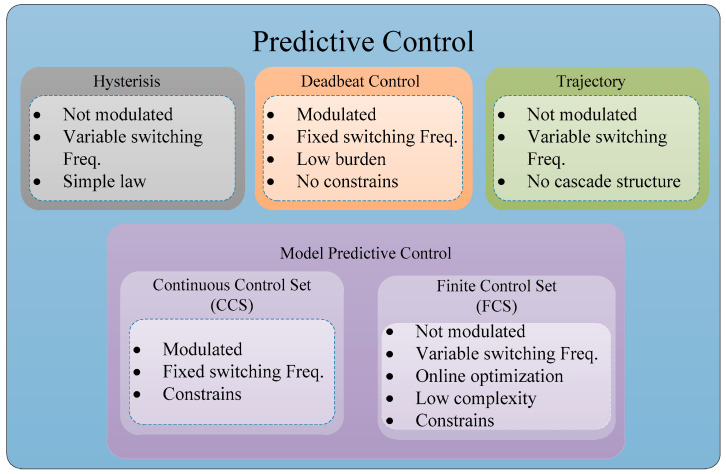
Model predictive control for power electronics.

Renewable energy sources are typically situated in areas with abundant natural resources, challenging the traditional assumption of a unidirectional power flow [1,2]. This shift has led to the development of distributed power systems, which are more complex and require advanced control mechanisms compared to conventional power systems [3]. Despite the valuable experience gained globally in the conventional energy sector, adapting to this new paradigm requires a re-evaluation of our existing infrastructure [4,5,6]. Additionally, the unique characteristics of renewable sources add layers of complexity. For example, solar energy is harvested as DC, wind turbines generate AC with variable frequencies depending on the wind speed, and similar variations are observed in wave energy and other renewable energy sources [7,8,9,10,11,12]. Consequently, achieving seamless integration of these diverse energy forms into power systems requires a meticulous energy conversion process [13,14]. Moreover, ensuring stability while maintaining grid supply voltage within the limits is crucial. Integrating renewable energies not only requires adjustments to infrastructure and control systems but also a sophisticated approach to energy conversion. As a result, static power converters become essential in renewable energy applications for delivering energy within the operational constraints of the AC mains [12,15]. However, static converters are nonlinear, have wide bandwidths, and are coupled systems. Consequently, control of these converters needs to be carefully designed [16,17]. To ensure effective power injection into the grid, precise control techniques are essential to regulate the internal variables of the power converters. Some of the most employed control strategies are Resonant Control [18], Nonlinear Feedback-Based Control [16], and model predictive control (MPC) [19], among others. Furthermore, the diverse range of applications for power converters necessitates robust yet flexible control techniques. MPC has garnered particular interest due to its straightforward formulation of the mathematical model through the physical laws governing electrical systems [20]. The chart in Figure 1 displays the predictive controllers commonly employed in power electronics. Of particular interest is model-based predictive control (MPC), which has been extensively utilized and is well documented in works [21,22,23,24,25,26]. MPC is noted for its outstanding dynamic performance and straightforward implementation. However, MPC is often associated with steady-state errors because it optimizes instantaneous values relative to a reference rather than the average value. This issue becomes more pronounced when there is significant uncertainty in the design parameters compared to the actual system values.

This paper proposes an enhanced MPC approach that retains the dynamic benefits of MPC while incorporating first-order state variable feedback to achieve zero steady-state error and optimize the eigenvalue placement. While some existing approaches have introduced state feedback into predictive control to enhance performance, our contribution addresses unique aspects. For example, [27] presented model predictive control with state feedback for a voltage-source converter with an LCL filter, where state feedback was used for control purposes such as damping and managing variable switching frequencies. In [28], predictive control was applied to direct torque control of an induction machine, using state feedback to improve the stator flux trajectories and torque while maintaining an acceptable switching frequency. In [29], output feedback was utilized to extend the operating range of a nonlinear system, and in [30], state feedback was employed in torque control to enhance the dynamic response and stability, similar to the objectives of this work. However, despite the introduction of state feedback in these contexts, the issues of achieving zero steady-state error in MPC and ensuring robustness under parameter variations have not been addressed, to the best of the authors’ knowledge, marking the main novelty of this contribution. Additionally, this study includes an analysis of robustness and sensitivity, emphasizing the advantages of this enhancement. The theoretical framework is validated through simulations and experimental tests.

## 2. The Power Converter Model

### 2.1. The Continuous Time Converter Model

**Figure 2:**
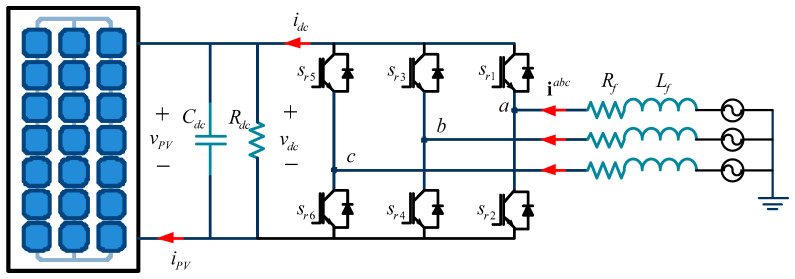
Three-phase power converter topology for injecting power into the main grid.

By applying Kirchhoff’s current and voltage laws, a mathematical model for a three-phase Active Front End (AFE) can be derived from Figure 2 [31]. This model describes the variations in current through the AC inductor and the voltage across the DC capacitor as follows:

(1) $$\frac{d}{dt}i^{abc}=-\frac{G_{ac}}{L_{f}}m^{abc}v_{dc}-\frac{R_{f}}{L_{f}}i^{abc}+\frac{1}{L_{f}}v_{s}^{abc}$$

and

(2) $$\frac{d}{dt}i^{abc}=-\frac{G_{ac}}{L_{f}}m^{abc}v_{dc}-\frac{R_{f}}{L_{f}}i^{abc}+\frac{1}{L_{f}}v_{s}^{abc}$$

where, respectively, **i***^abc^* is the three-phase current, **v***_s_^abc^* is the three-phase grid tension, **m***^abc^* is a three-phase modulator signal, *v_dc_* is the DC link voltage, and *G_ac_*, *R_f_*, *L_f_*, and *C_dc_* are constant values that describe the converter gain, resistance, and inductance of the first-order filter and the capacitance of the DC link, respectively. Finally, *i_pv_*(*v_dc_*) is the current produce due to the photovoltaic effect in the solar panel.

To implement classical control techniques for the analysis and design of the control system, an equivalent power invariant transformation [32] is applied to the system, defined by

(3) $$T_{abc-dq0}=\sqrt{\frac{2}{3}}\left[\begin{array}{ccc} \sin\left(\omega t\right) & \sin\left(\omega t-\frac{2\pi }{3}\right) & \sin\left(\omega t+\frac{2\pi }{3}\right)\\ \cos\left(\omega t\right) & \cos\left(\omega t-\frac{2\pi }{3}\right) & \cos\left(\omega t+\frac{2\pi }{3}\right) \end{array}\right]$$

**Figure 3:**
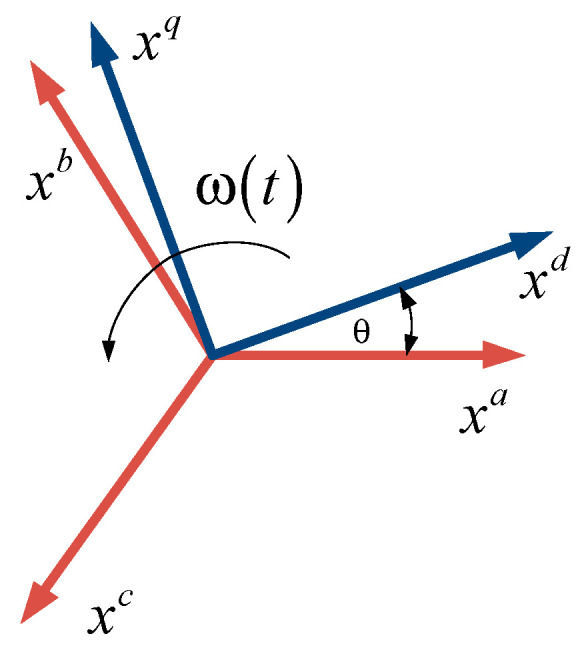
Park’s transform.

Park’s transform, presented in (3), projects the static three-phase reference system onto a synchronous rotating axis, as shown in Figure 3. It is noteworthy that (3) is time-dependent, and its derivative is given by

(4) $$\frac{d}{dt}\left\{T_{dq0-abc}\right\}=T_{dq0-abc}W$$

with the matrix

(5) $$W=\left(\begin{array}{cc} 0 & -\omega \\ \omega & 0 \end{array}\right)$$

where ω is the grid’s angular frequency. Thus, by applying this transformation to (1) and (2), a continuous representation of the three-phase *ac* system is attained as

(6) $$\frac{d}{dt}i^{dq0}=\frac{1}{L_{f}}{v_{s}}^{dq0}-\frac{R_{f}}{L_{f}}i^{dq0}-Wi^{dq0}-\frac{1}{L_{f}}\left(G_{ac}m^{dq0}v_{dc}\right)$$

(7) $$\frac{d}{dt}v_{dc}=\frac{i_{pv}(v_{dc})}{C_{dc}}+\frac{G_{ac}}{C_{dc}}{\left(m^{dq}\right)}^{T}i^{dq}-\frac{v_{dc}}{R_{dc}C_{dc}}$$

Assuming that (1) represents a balanced system, (6) and (7) are separated into direct and quadrature components, leaving behind the “0” component since all of them are zero vectors because this is considered a balanced system. Thus, the system

(8) $$\frac{d}{dt}i^{d}=\frac{1}{L_{f}}v_{s}^{d}-\frac{R_{f}}{L_{f}}i^{d}+\omega i^{q}-\frac{1}{L_{f}}G_{ac}m^{d}v_{dc}$$

(9) $$\frac{d}{dt}i^{q}=\frac{1}{L_{f}}v_{s}^{q}-\frac{R_{f}}{L_{f}}i^{q}-\omega i^{d}-\frac{1}{L_{f}}G_{ac}m^{q}v_{dc}$$

(10) $$\frac{d}{dt}v_{dc}=\frac{i_{pv}(v_{dc})}{C_{dc}}+\frac{G_{ac}}{C_{dc}}\left(m^{d}i^{d}+m^{q}i^{q}\right)-\frac{v_{dc}}{R_{dc}C_{dc}}$$

describes the dynamic converter model.

### 2.2. The Discrete Time Converter Model

As the predictive controller is a discrete time algorithm, a discrete approximation to the system model is obtained. Consider the Forward Euler Approximation [33]:

(11) $$\frac{d}{dt}x\approx \frac{x\left(k+1\right)-x\left(k\right)}{T_{s}}$$

where *x*(*k*) is the present value of the state variable, *x*(*k* + 1) is the value of the state at the next time step, and *T_s_* is the sampling period [34]. Thus, discretizing (8) and (9), a discrete state space is obtained:

(12) $$i^{dq}(k+1)=Fi^{dq}(k)+Gm^{dq}(k)+Ev_{s}^{dq}(k)$$

(13) $$y=Ci^{dq}(k)$$

with

(14) $$F=\left[\begin{array}{cc} 1-\frac{R_{f}T_{s}}{L_{f}} & \omega T_{s}\\ -\omega T_{s} & 1-\frac{R_{f}T_{s}}{L_{f}} \end{array}\right]$$

(15) $$G=\left[\begin{array}{cc} \frac{-G_{ac}T_{s}V_{dc}}{L_{f}} & 0\\ 0 & \frac{-G_{ac}T_{s}V_{dc}}{L_{f}} \end{array}\right]$$

(16) $$E=\left[\begin{array}{cc} \frac{T_{s}}{L_{f}} & 0\\ 0 & \frac{T_{s}}{L_{f}} \end{array}\right]$$

and **C** is an order 2 identity matrix. The discrete dynamic behavior of (12) can be described as the future output of the system one step forward. Thus, the input **m***^dq^*(*k*) that generates such output can be obtained. Note that the voltage *V_dc_* is considered a constant value, which is reasonable given its dynamics is usually much slower when compared to that of the current.

## 3. The Predictive Current Control Loop

### 3.1. Controller Output Prediction

A prediction of the controller output can be obtained from (12) as

(17) $$m^{dq}(k)=G^{-1}\left(i^{dq}(k+1)-Fi^{dq}(k)-Ep^{dq}(k)\right)$$

Note that for **m***^dq^*(*k*) to be bounded, with *det*{**G**} ≠ 0, by inspecting (15), it is noticeable that given *G_ac_*, *T_s_*, and *L_f_*, are known to be constant, it is just up to the DC voltage to not be null for the output **m***^dq^*(*k*) to be defined. Moreover, the greater *V_dc_*, the smaller the expected controller output.

The computation delay must be compensated for with a second prediction; then,

(18) $$i^{dq}(k+2)=Fi^{dq}(k+1)+Gm^{dq}(k+1)+Ev_{s}^{dq}(k+1)$$

It is noteworthy that **i***^dq^*(*k* + 1), from (12), when replaced in (18), gives

(19) $$i^{dq}(k+2)=F^{2}i^{dq}(k)+U_{o}\left[\begin{array}{c} m^{dq}(k+1)\\ m^{dq}(k) \end{array}\right]+P_{o}\left[\begin{array}{c} v_{s}^{dq}(k+1)\\ v_{s}^{dq}(k) \end{array}\right]$$

with **U***_o_* = [**G FG**] and **P***_o_* = [**E FE**]. Note that **U***_o_* is the controllability matrix, and if *rank* (**U***_o_*) = *n*, with *n* being the order of the system, the system is controllable.

From (19), the first and second predictions can be derived as

(20) $$u^{*}=U_{o}^{T}{\left(U_{o}U_{o}^{T}\right)}^{-1}\left(i_{ref}^{dq}-F^{2}i^{dq}(k)-P_{o}p\right)$$

Given that **U***_o_* has dimensions of *n ×* 2*n* and therefore is non-invertible, the pseudoinverse is applied instead, as shown in (20), where u* = [**m***^dq^*(*k* + 1) **m***^dq^*(*k*)]*^T^* is a vector that contains the first and second predictions of the output, **p** = [**v***_s_^dq^*(*k* + 1) **v***_s_^dq^*(*k*)]*^T^* the vector that contains the disturbance (grid voltage), and **i***^dq^*(*k* + 2) the desired system output. The predictions of the control effort can be extended even further as

(21) $${u_{N}}^{*}=U_{N}^{+}\left(i_{ref}^{dq}-F^{N}i^{dq}(k)-P_{N}p_{N}\right)$$

with **U***_N_*^+^ = **U***_N_^T^*(**U***_N_* **U***_N_^T^*)^−1^ the pseudoinverse of **U***_N_* = [**G FG**… **F***^N^*^−1^**G**], u* = [**m***^dq^*(*k* + *N*) **m***^dq^*(*k* + *N* − 1)… **m***^dq^*(*k*)]*^T^* the predicted output, **P***_o_* = [**E FE**… **F***^N^*^−1^**E**] the disturbance matrix, and **p***_N_* = [**v***_s_^dq^*(*k* + *N*) **v***_s_^dq^*(*k* + *N* − 1)… **v***_s_^dq^*(*k*)]*^T^* the predicted disturbances. Here, subindex *N* indicates the prediction horizon. It is clear from (19)–(21) that the control effort is determined by the plant parameters. It is important to mention that the parameters in the controller and system matrices **F**, **G**, and **E** used in the algorithm are exact and the same, as shown in Table 1.

**Figure 4:**
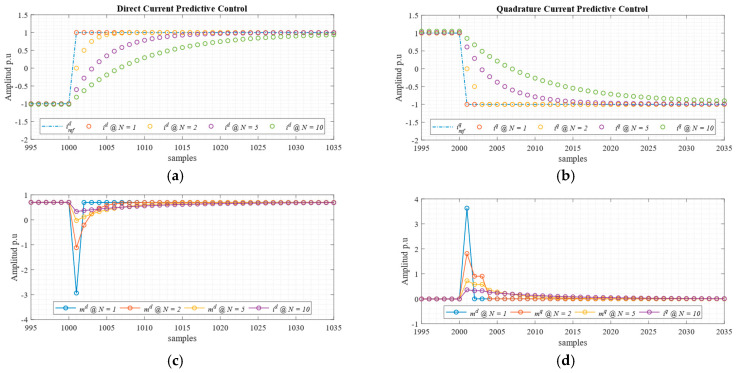
Predictive control horizon comparison. (**a**,**b**) Direct and quadrature current dynamic responses, respectively; (**c**,**d**) direct and quadrature modulators, respectively.

In Figure 4, a comparison of the responses of both sampled current control loops, and control of their efforts (modulators), when using different prediction horizons is shown. Clearly, when the horizon is the shortest (*N* = 1), the highest controller output is applied; thus, the system reaches a steady state in one sample. In extending the prediction horizon, as presented in (21), a more relaxed response is obtained, and therefore a reduced control effort is calculated.

### 3.2. Loop Robustness and Sensibility

To design a robust controller, parameter uncertainty needs to be taken into account. Let **F***_c_*, **G***_c_*, and **E***_c_* be evolution, control, and disturbance matrices with uncertain parameters. Then, from (20), a control law for a horizon *N* = 2 can be established as

(22) $${u_{c}}^{*}=U_{c}^{T}{\left(U_{c}U_{c}^{T}\right)}^{-1}\left(i_{ref}^{dq}-{F_{c}}^{2}i^{dq}(k)-P_{c}p\right)$$

with **U***_c_* = [**G***_c_* **F***_c_***G***_c_*] and **P***_c_* = [**E***_c_* **F***_c_***E***_c_*]. From (12) and (18), let us establish the relationship that describes the evolution of the system and its prediction as

(23) $$i^{dq}(k+2)=F^{2}i^{dq}(k)+U_{o}u^{*}+P_{o}p$$

Substituting (22) into (23), the evolution of the system in terms of the reference and the controller parameters

(24) $$i^{dq}(k+2)=F^{2}i^{dq}(k)+U_{o}\left({U_{c}}^{+}\left(i_{ref}^{dq}-{F_{c}}^{2}i^{dq}(k)-P_{c}p\right)\right)+P_{o}p$$

Then, (24) is arranged as

(25) $$i^{dq}(k+2)=F_{\sigma }i^{dq}(k)+G_{\sigma }i_{ref}^{dq}+E_{\sigma }p$$

where

(26) $$F_{\sigma }=F^{2}-U_{o}{U_{c}}^{+}F_{c}^{2}$$

(27) $$G_{\sigma }=U_{o}{U_{c}}^{+}$$

(28) $$E_{\sigma }=P_{o}-U_{o}{U_{c}}^{+}P_{c}$$

**Figure 5:**
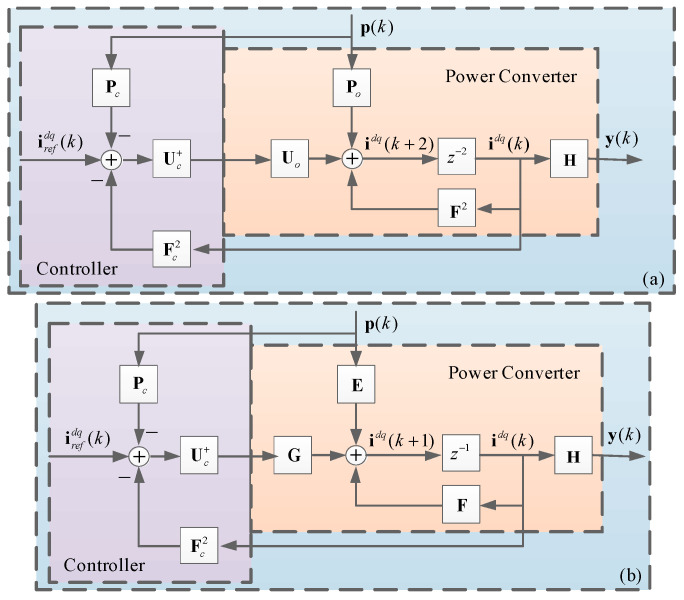
Predictive control loop: (**a**) control loop as shown in (19); (**b**) control loop as shown in (18).

**Figure 6:**
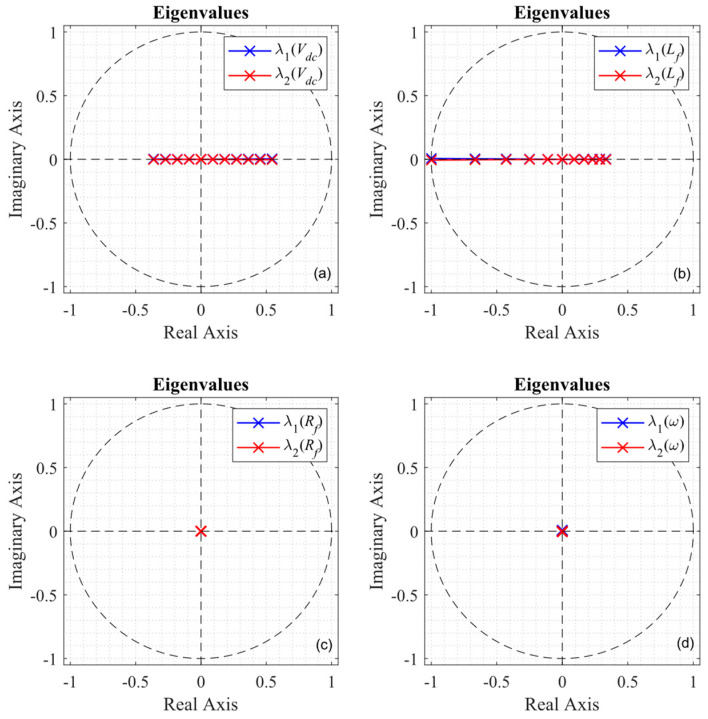
Predictive control eigenvalues under parameter variation: (**a**) changes in *V_dc_*; (**b**) changes in L_f_; (**c**) changes in R_f_; (**d**) changes in ω.

This results in a closed-loop system, as shown in Figure 5a. Finally, the eigenvalues are dictated by **F**_σ_, which depends on the values of the predictive control parameters, thus revealing the stability and robustness of the predictive controller. By solving the characteristic equation

(29) $$\left|\lambda I-F_{\sigma }\right|=0$$

the eigenvalues as a function of the parameter variation are shown in Figure 6. A system with these exact parameters would see a second-order time delay (*z*^−2^), meaning that the two eigenvalues resulting from the solution of (29) would be zero. The eigenvalues for a shift in the DC voltage of ±50% are shown in Figure 6a, changing the behavior mostly along the real axis and producing a slower response. However, it is clear from Figure 6b that the control system is most sensitive to the inductor value, even becoming unstable for the ±50% variations proposed. On the other hand, uncertainty in the line resistance and grid frequency have little to no effect on the position of the eigenvalues, showing robustness for a ±50% shift, as shown in Figure 6c,d.

### 3.3. Steady-State Response

A small yet powerful analysis of the steady-state response is a necessary step to understand and improve the controller. From (25) and (13), it is possible to obtain an expression such that by applying the ℤ{·} transform, we obtain

(30) $$z^{2}i^{dq}(z)=F_{\sigma }i^{dq}(z)+G_{\sigma }i_{ref}^{dq}(z)+E_{\sigma }p(z)$$

and

(31) $$y(z)=Ci^{dq}(z)$$

Then, by substituting the state vector **i***^dq^*(*z*) from (30) into (31), the system’s output as a function of the reference vector is **i***_ref_^dq^*(*z*)

(32) $$y(z)=C{\left(z^{2}I-F_{\sigma }\right)}^{-1}G_{\sigma }i_{ref}^{dq}(z)$$

The steady-state response of (32) is

(33) $$\underset{k\to \infty }{\lim}y(k)=\underset{z\to 1}{\lim}\left(1-z^{-1}\right)y(z)$$

leading to

(34) $$\underset{k\to \infty }{\lim}y(k)=\underset{z\to 1}{\lim}\left(1-z^{-1}\right)C{\left(I-F_{\sigma }\right)}^{-1}G_{\sigma }i_{ref}^{dq}(z)$$

For constant values of $i_{ref}^{dq}$ (meaning the step response), (34) stays as

(35) $$\underset{k\to \infty }{\lim}y(k)=C{\left(I-F_{\sigma }\right)}^{-1}G_{\sigma }$$

Substituting (26) and (27) into (35), we derive

(36) $$\underset{k\to \infty }{\lim}y(k)=C{\left(I-F^{2}+U_{o}U^{+}F_{c}^{2}\right)}^{-1}U_{o}{U_{c}}^{+}$$

meaning the output of the system will be equal to the reference (no steady-state error) when the parameters that build the control law and those of the system are identical. However, this is not a realistic assumption since parameters vary from time to time and with temperature, current, voltage, and other disturbances.

**Figure 7:**
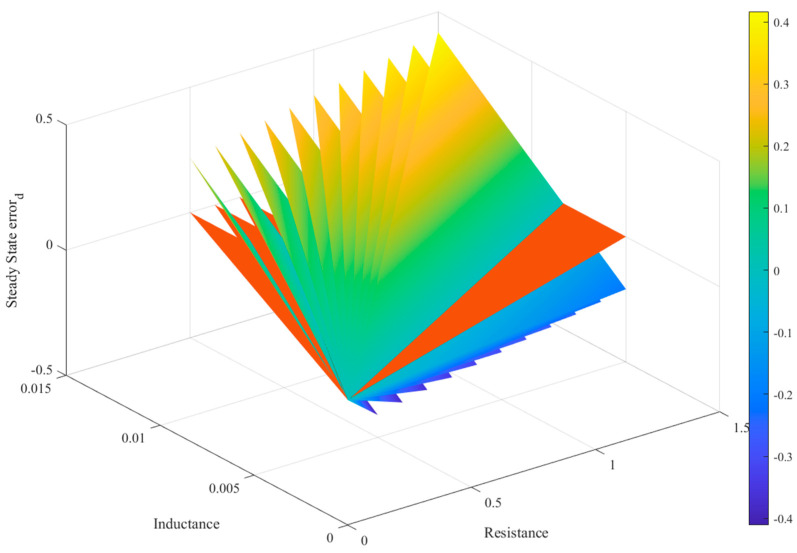
Steady-state error for variable system parameters.

The graph presented in Figure 7 shows the steady-state error of the direct component of the current under variation in the grid filter parameters (*R_f_* and *L_f_*). The red-colored plane indicates the zero-error reference. It is notable that the variation in the system’s parameters generates planes that cross the reference, meaning that exact knowledge of the parameters is not necessarily necessary, but that of the values of the elements of the matrices **F**^2^, **U***_o_* and **F***_c_*^2^, **U***_c_*, respectively, are.

### 3.4. Lyapunov Stability Analysis

Consider the autonomous part of the control system in (25) as

(37) $$x(k+1)=F_{a}x(k)$$

where

(38) $$F_{a}=\left[\begin{array}{cc} 0 & I\\ F_{\sigma } & 0 \end{array}\right]$$

and the vector **x**(*k*) = [**x_1_**(*k*) **x_2_**(*k*)]*^T^* = [**i***^dq^*(*k*) **i***^dq^*(*k* + 1)]*^T^*. Notice that external input and disturbances are not considered. Then, the system is said to be asymptotically stable at the origin if for any symmetric positive definite matrix **M**, a symmetric positive definite matrix **P** exists that satisfies the Lyapunov equation

(39) $$F_{a}^{T}PF_{a}-P=-M$$

By designing the Lyapunov function [35,36] as

(40) $$V(x)=x^{T}Px$$

then

(41) V(x)>0 for any x≠0

and

(42) V(0)=0

The difference

(43) ΔV(x)=V(x(k+1))−V(x(k))

Then, by substituting (37) into (43),

(44) $$\Delta V(x)=x^{T}(k)F_{a}^{T}PF_{a}x(k)-x^{T}(k)Px(k)$$

by arranging

(45) $$\Delta V(x)=x^{T}(k)\left(F_{a}^{T}PF_{a}-P\right)x(k)$$

Finally, from (39), we obtain the variation in the Lyapunov function as

(46) $$\Delta V(x)=-x^{T}(k)Mx(k)$$

As **M** was defined as a positive definite matrix, then (46) is negative definite. Thus, the system is asymptotically stable at the origin. Let **M** be defined arbitrarily as an identity matrix **I** of order 2*n* × 2*n*. Then, (39) turns into

(47) $$F_{a}^{T}PF_{a}-P=-I$$

Solving (47) for **P** derives

(48) $$P=\left[\begin{array}{cccc} 2 & 0 & 0 & 0\\ 0 & 2 & 0 & 0\\ 0 & 0 & 1 & 0\\ 0 & 0 & 0 & 1 \end{array}\right]$$

which is a symmetrical positive definite matrix. Then, the autonomous system of (37) is asymptotically stable around the origin.

## 4. Integral State Feedback Support

**Figure 8:**
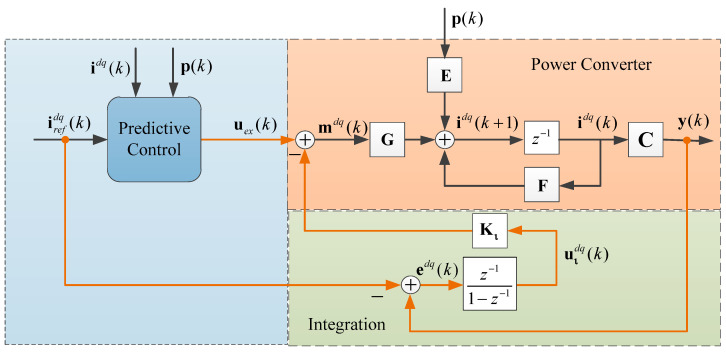
Integral feedback.

As mentioned in the previous section, parameter variations impose an undesired tracking error, and the absence of memory in the control law means that the controller calculates the next *N* output without considering the past output. Thus, it does not allow this steady-state error to be corrected, as established in (36). In Figure 5, the proposed predictive control loop does not calculate the error, being incapable of correcting it. To provide memory to the system and define desirable dynamic and static characteristics, the control loop presented in Figure 8 is proposed. It is considered that the controller designed to support static behavior is not compensated for in the predictive algorithm, so there is a parallel influence on the control signal.

As discussed earlier, parameter variations lead to undesired tracking errors due to the control law’s lack of memory, meaning the controller computes the next output without considering past output. Consequently, it fails to correct steady-state errors, as indicated in Equation (36). In Figure 5, the suggested predictive control loop does not calculate the error and thus cannot correct it. To address this issue and establish the desired dynamic and static characteristics, Figure 8 introduces a control loop that incorporates memory into the system. It is essential to note that the controller designed to support static behavior is not compensated for in the predictive algorithm, thereby exerting a parallel influence on the control signal.

### 4.1. Discrete Integration

Let us consider the rectangular approximation of the integral as

(49) $$u(t)=\int x(t)dt\approx \sum x(k)T_{s}$$

which can be expressed as a difference equation as

(50) $$u_{\iota }^{dq}(k)=u_{\iota }^{dq}(k-1)+e^{dq}(k)$$

Thus, the discrete transfer function, considering the computational delay, is

(51) $$\frac{u_{\iota }^{dq}(z)}{e^{dq}(z)}=\frac{z^{-1}}{1-z^{-1}}$$

and is shown in the simulation diagram in Figure 8. Shifting (50) forward by one sample period (*k* → *k* + 1) gives us

(52) $$\begin{array}{l} u_{\iota }^{dq}(k+1)=u_{\iota }^{dq}(k)+e^{dq}(k)\\ \;\;\;\;\;\;\;\;\;\;\;\;\;\;\;\;=u_{\iota }^{dq}(k)+(Ci^{dq}(k)-i_{ref}^{dq}(k)) \end{array}$$

where **e***^dq^*(*k*) is the error of the system at time *k*, and **u_ι_***^dq^*(*k*) is the discrete integrator output. Then, the modulator signal

(53) $$m^{dq}(k)=u_{ex}^{dq}-K_{\iota }u_{\iota }^{dq}(k)$$

When considering both predictive control and integral feedback, as shown in Figure 8, extended state feedback is obtained. In order to study and modify the dynamic response of the control loop, it is necessary to introduce the external input **u***_ex_^dq^*(*k*) = **u**_c_^*^ (*k*). With the predictive controller output from (22), then the modulator signal is given by

(54) $$m^{dq}(k)=u_{c}^{*}(k)-K_{\iota }u_{\iota }^{dq}(k)$$

where **K_ι_** is a symmetric positive definite matrix for the feedback of the extended states. Given that just the first prediction is considered, we use the matrices **U***_c_* = **G***_c_* and **P***_c_* = **E***_c_*. Substituting (54) into (18) obtains

(55) $$i^{dq}(k+1)=Fi^{dq}(k)+G\left(u_{1}^{*}(k)-K_{\iota }u_{\iota }^{dq}(k)\right)+E_{\iota }p^{dq}$$

Thus, considering (55) as the state space system and (52) as a co-state from the discrete integration, the extended state space representation of the system with integral feedback therein

(56) $$\psi^{dq}(k+1)=F_{\psi }\psi^{dq}(k)+E_{\psi }p^{dq}(k)-I_{\psi }i_{ref}^{dq}(k)$$

with the constant matrices

(57) $$F_{\psi }=\left[\begin{array}{cc} F-GG_{c}^{-1}F_{c} & -GK_{\iota }\\ C & I \end{array}\right]$$

(58) $$E_{\psi }={\left[\begin{array}{cc} E-GG_{c}^{-1}E_{c} & 0 \end{array}\right]}^{T}$$

(59) $$I_{\psi }={\left[\begin{array}{cc} GG_{c}^{-1} & I \end{array}\right]}^{T}$$

Also, with **ψ***^dq^*(*k*) = [**i***^dq^*(*k*) **u_ι_***^dq^*(*k*)]*^T^* as the extended discrete state variable, **u***_ex_^dq^*(*k*) is an external and independent input (in this case, from the predictive controller). The new eigenvalues of the extended systems are in **F**_ψ_. Then, by modifying the values of the feedback matrix **K_ι_**, the dynamic response of the closed-loop system will change. The expression in (57) is modified as

(60) $$F_{\psi }=\left[\left[\begin{array}{cc} A & 0\\ C & I \end{array}\right]-\left[\begin{array}{c} G\\ 0 \end{array}\right]\left[\begin{array}{cc} \Delta k & K_{\iota } \end{array}\right]\right]$$

with

(61) $$A=F-GG_{c}^{-1}F_{c}+G_{c}\Delta k$$

where Δ**k** is a predictive controller compensation term for the state feedback. In this manner, the eigenvalues of the system can be modified at will. Now, we can define

(62) $$F_{K}=\left[\begin{array}{cc} A & 0\\ C & I \end{array}\right]$$

(63) $$G_{K}={\left[\begin{array}{cc} G & 0 \end{array}\right]}^{T}$$

(64) $$K=\left[\begin{array}{cc} \Delta k & K_{\iota } \end{array}\right]$$

This notation will be useful for the following eigenvalue placement.

### 4.2. Optimal Eigenvalue Placement

The values of the matrix **K_ι_** will change the dynamic and static responses of the system; thus, in order to place the eigenvalues of the closed-loop system optimally, a discrete cost functional is proposed in terms of the expected energy of the states and the controller output as

(65) $$J={\left(\psi^{dq}(N)\right)}^{T}H\left(\psi^{dq}(N)\right)+{\sum_{k=0}^{N-1}{\left(\psi^{dq}(k)\right)}^{T}Q\left(\psi^{dq}(k)\right)+\left(u_{\iota }^{dq}(k)\right)}^{T}R\left(u_{\iota }^{dq}(k)\right)$$

where **H**, **Q**, and **R** are constant symmetric positive definite matrices. To find a solution that minimizes the cost functional, let us consider the cost functional for the *N* time instant

(66) $$\begin{array}{ll} J_{N,N}(\psi (N)) & ={\left(\psi^{dq}(N)\right)}^{T}H\left(\psi^{dq}(N)\right)\\ & ={\left(\psi^{dq}(N)\right)}^{T}P(0)\left(\psi^{dq}(N)\right) \end{array}$$

where **P**(0) is an arbitrarily defined dynamic matrix that gives relative weight to the steady-state response of the state vector **ψ***^dq^*(*N*) = [**i***^dq^*(*N*) **u_ι_***^dq^*(*N*)]*^T^*. Then, the cost functional for *N −* 1 is

(67) $$\begin{array}{ll} J_{N-1,N}\left(\psi (N-1),u_{\iota }^{dq}(N-1)\right)= & {\left(\psi (N)\right)}^{T}P(0)\left(\psi (N)\right)+{\left(\psi (N-1)\right)}^{T}Q\left(\psi (N-1)\right)+\cdots \\ & {\left(u_{\iota }^{dq}(N-1)\right)}^{T}R\left(u_{\iota }^{dq}(N-1)\right). \end{array}$$

We substitute (56) as a function of *N* into (67) and arrange the result:

(68) $$\begin{array}{ll} J_{N-1,N}\left(\psi (N-1),u_{\iota }^{dq}(N-1)\right)= & {\left(\psi (N-1)\right)}^{T}\left(F_{K}^{T}P(0)F_{K}+Q\right)\left(\psi (N-1)\right)+\cdots \\ & {\left(u_{\iota }^{dq}(N-1)\right)}^{T}\left(G_{K}^{T}P(0)G_{K}+R\right)\left(u_{\iota }^{dq}(N-1)\right)+\cdots \\ & {\left(\psi (N-1)\right)}^{T}\left(F_{K}^{T}P(0)G_{K}\right)\left(u_{\iota }^{dq}(N-1)\right)+\cdots \\ & {\left(u_{\iota }^{dq}(N-1)\right)}^{T}\left(G_{K}^{T}P(0)F_{K}\right)\left(\psi (N-1)\right). \end{array}$$

Then, to find the controller output that minimizes (68) such that

(69) $$J_{N-1,N}\left(\psi (N-1)\right)=\underset{u_{\iota }^{dq}(N-1)}{\min}\left\{\ldots \right\}$$

it is necessary to find the minimum as the partial derivative equal to zero such that

(70) $$\frac{\partial }{\partial u_{\iota }^{dq}}J_{N-1,N}\left(\psi (N-1),u_{\iota }^{dq}(N-1)\right)=0$$

Then, solving for **u_ι_***^dq^*(*N* − 1),

(71) $$u_{\iota }^{dq}(N-1)={\left(G_{K}^{T}P(0)G_{K}+R\right)}^{-1}G_{K}^{T}P(0)F_{K}\psi (N-1)$$

or

(72) $$u_{\iota }^{dq}(N-1)=-K(N-1)\psi (N-1)$$

the optimal control output is a function of the extended states of the system and the dynamic matrix **K_ι_**(*N* − 1), which will place the eigenvalues of the systems for the optimal response of the closed-loop system. The dynamic behavior can be expressed in terms of the *j*-th iteration as

(73) $$K(N-j)={\left[R+G_{K}^{T}P(j-1)G_{K}\right]}^{-1}G_{K}^{T}P(j-1)F_{K}$$

And the dynamic matrix **P**(*j*) is updated as

(74) $$\begin{array}{ll} P(j)= & {\left[F+GK(N-j)\right]}^{T}P(j-1)\left[F+GK(N-j)\right]+\ldots \\ & \ldots +K^{T}RK(N-j)+Q. \end{array}$$

**Figure 9:**
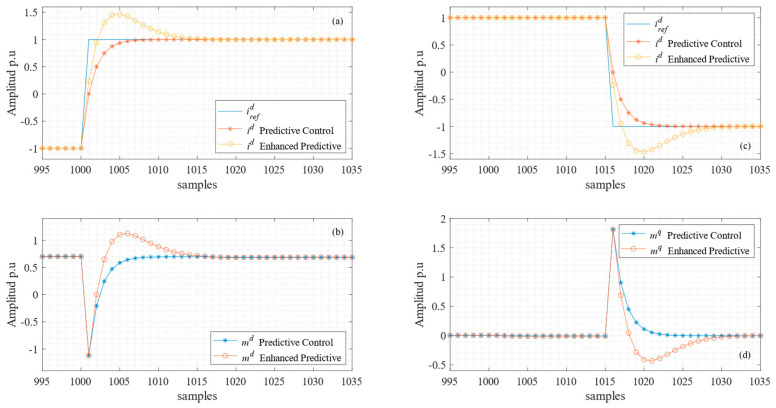
Predictive control vs. enhanced predictive control linear dynamic response. (**a**) Direct current response comparison i^d^; (**b**) direct component of the modulator signal m^d^; (**c**) quadrature current comparison i^q^; (**d**) quadrature component of the modulation signal m^q^.

In arbitrarily choosing the matrices **H**, **R**, and **Q**, it was possible to shape the linear response, as shown in Figure 9, considering the exact knowledge of the system parameters from Table 1. The predictive controller and its enhanced response for direct and quadrature currents are shown in Figure 9a and Figure 9c, respectively. There is a remarkable difference in the overshoot of both responses, almost 25% for the enhanced controller. However, the settling time remains mostly unchanged. Both components of the controller output are shown in Figure 9b,c, and the steady-state values of the modulator signals are the same in both cases.

### 4.3. Steady-State Response

To show the improvement in the steady-state response due to the integral support, we apply the final value theorem in the discrete frequency domain. Applying the *ℤ*{·} transform to (55) and solving for **i***^dq^*(*z*) and substituting it into the error equation,

(75) $$e^{dq}(z)=Ci^{dq}(z)-i_{ref}^{dq}(z)$$

Solving again for **i***^dq^*(*z*), we obtain

(76) $$i^{dq}(z)=-{\left(zI-F-GG_{c}^{-1}F_{c}\right)}^{-1}GK_{\iota }u_{\iota }^{dq}(z)$$

Then, applying the *ℤ*{·} transform to (52), solving for **u_ι_***^dq^*(*z*) and substituting it into (76), and then solving for **e***^dq^*(*z*) derives

(77) $$e^{dq}(z)=-\left(zI-I\right){\left[\begin{array}{l} \left(zI-I\right)+\cdots \\ C{\left(zI-F-GG_{c}^{-1}F_{c}\right)}^{-1}GK_{\iota } \end{array}\right]}^{-1}i_{ref}^{dq}(z)$$

Ultimately, evaluating the limit of (77), for *z* → 1, the final value of the error is obtained as

(78) $$\underset{z\to 1}{\lim}\left(1-z^{-1}\right)e^{dq}(z)=0$$

**Figure 10:**
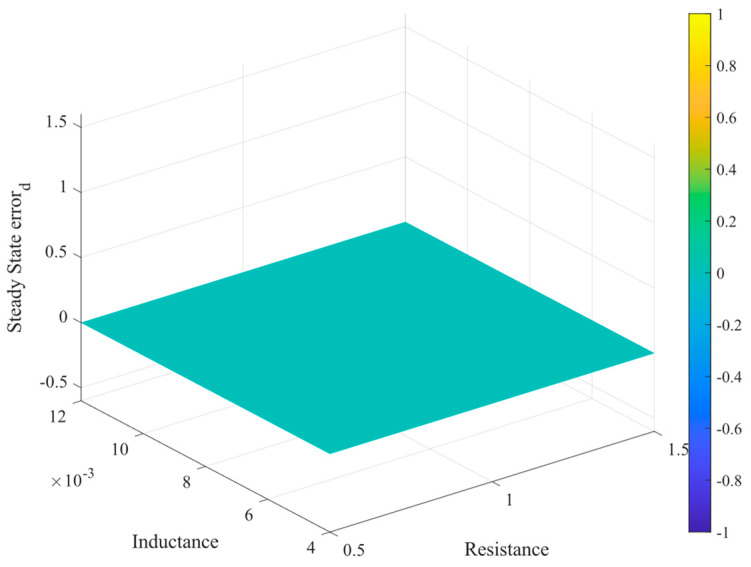
Steady-state error of the enhanced predictive controller for parameter variation.

Therefore, the enhanced static characteristics of the system are clearly demonstrated. Zero steady-state error can be attained without requiring precise knowledge of the system’s parameters (within the range of robustness). As shown in Figure 10, where ±50% uncertainty is introduced into the system parameters, it is noteworthy that the integral feedback provides greater flexibility in the loop design while maintaining a rapid dynamic response, as illustrated in Figure 9.

### 4.4. Robustness and Sensitivity

**Figure 11:**
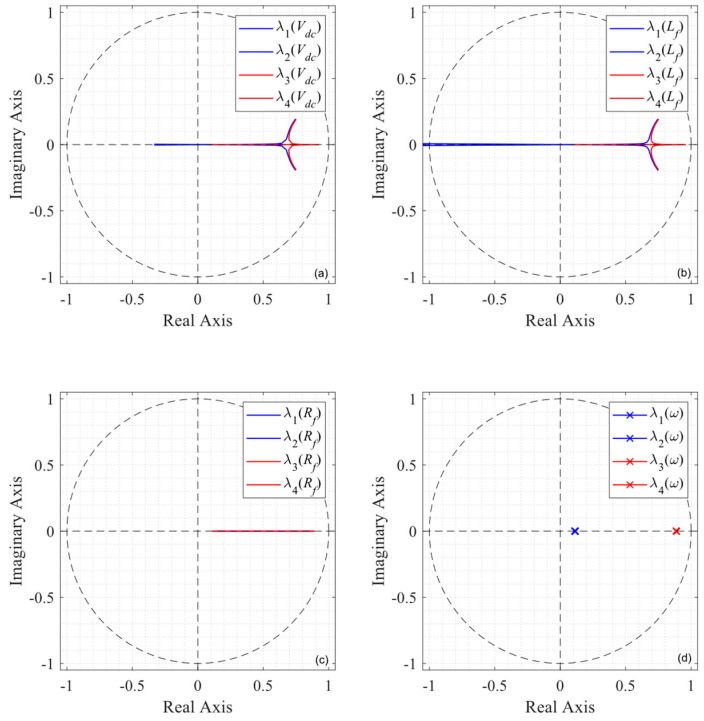
Enhanced predictive control eigenvalues under parameter variation: (**a**) changes in V_dc_; (**b**) changes in L_f_; (**c**) changes in R_f_; (**d**) changes in ω.

The addition of the integrative action provides the linear system with better reference tracking. However, as shown in Figure 11, when compared to Figure 6, the entire system is more sensitive to parameter variation or uncertainty. The effect of the DC voltage on the eigenvalues is shown in Figure 11a; the dynamic behavior of system will vary, but its stability is not compromised. Note that in the case when the inductor of the system differs by more than ±50% from its expected value (the value programmed in the controller), the eigenvalues leave the region of stability, as shown in Figure 11b. In the case of the resistor in Figure 11c, it is observable that region where the eigenvalues are located is extended, showing much more sensitivity, in contrast to Figure 6c. It is noteworthy that the linear system (as analyzed) is not affected by the grid frequency, as depicted in Figure 11d. However, this analysis only shows the behavior of the eigenvalues from a control system’s point of view and does not consider the unsynchronized interaction of the converter with the grid, which may greatly affect the entire stability of the topology [37].

## 5. Active and Reactive Power Control Loops

The design of a stable and robust predictive current control loop has been fully developed. The direct and quadrature current references are obtained from the active and reactive power control, respectively. To provide power from the PV array to the grid, a power control loop needs to be designed properly. In this document, a PI controller is implemented.

### 5.1. Plant Modeling

To define the PI controller gains, a mathematical model and eventually a transfer function for power control are necessary. Let us consider the expression for the continuous time representation of the energy in the DC link capacitor as

(79) $$E_{C_{dc}}(t)=C_{dc}{\left(v_{dc}(t)\right)}^{2}/2$$

Then, the power is obtained by deriving (79) and applying the Laplace transform ℒ{·}. A transfer function in the Laplace domain that relates the DC link capacitor power and the DC voltage squared can be obtained as

(80) $${\left(v_{dc}(s)\right)}^{2}/P_{dc}(s)=2/C_{dc}s$$

### 5.2. The DC Voltage Controller

**Figure 12:**
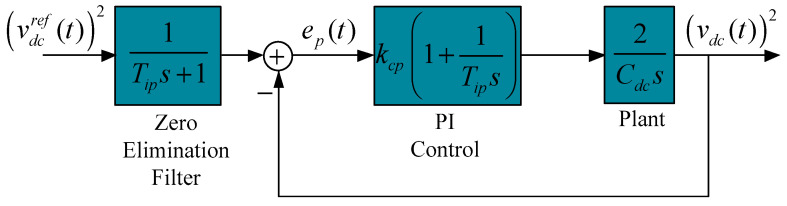
Simplified power control loop.

The closed-loop transfer function as shown in the simplified scheme in Figure 12 is given by

(81) $$H_{clp}(s)=\frac{2k_{cp}/C_{dc}T_{ip}}{s^{2}+2k_{cp}s/C_{dc}+2k_{cp}/C_{dc}T_{ip}}$$

From the general transfer function of a second-order system, the values of the PI controller gains can be obtained as

(82) $$k_{cp}=\xi \omega_{n}C_{dc}$$

(83) $$T_{ip}=2k_{cp}/C_{dc}\omega_{n}^{2}$$

where ξ is the dumping factor, ω*_n_* = 4/*T*_ss_ the natural frequency of the system, and *T*_ss_ is the desired settling time.

The discrete implementation of the designed PI controller is given by the following discrete equation:

(84) $$u_{p}(k)=u_{p}(k-1)+q_{0}e_{p}(k)+q_{1}e_{p}(k-1)$$

with

(85) $$q_{0}=k_{cp}(1+T_{s}/T_{ip})$$

(86) $$q_{1}=-k_{cp}$$

### 5.3. Current Reference

**Figure 13:**
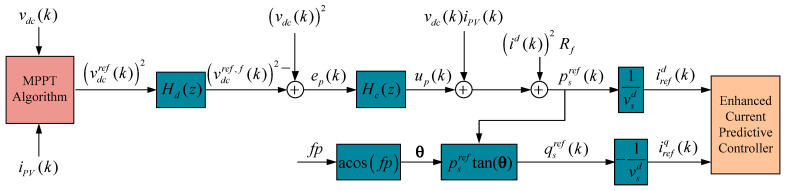
Detailed power control loop.

From the power balance, shown in Figure 13, the power is defined:

(87) $$P_{s}^{ref}=P_{pv}+P_{C_{dc}}+P_{RL}$$

where $P_{s}^{ref}$ is the desired power in the grid, *P_pv_* is the power provided by the PV array, $P_{C_{dc}}$ is the power in the DC link capacitor, and *P_RL_* is the dissipated power in the *RL* filter.

The relationship of the direct and quadrature currents in terms of the desired active and reactive power is given by

(88) $$i_{ref}^{d}=\left(P_{s}^{ref}v_{s}^{d}+Q_{s}^{ref}v_{s}^{q}\right)/{\left\|v_{s}^{dq}\right\|}^{2}$$

(89) $$i_{ref}^{q}=\left(P_{s}^{ref}v_{s}^{q}-Q_{s}^{ref}v_{s}^{d}\right)/{\left\|v_{s}^{dq}\right\|}^{2}$$

where $Q_{s}^{ref}$ is the reactive power. Considering that the *dq* transform is synchronized to the grid voltage, the quadrature component of the grid voltage is zero. Then, the simplified expressions for (88) and (89) are shown in the block diagram from Figure 13.

## 6. Results

The results presented in the following section, including the simulations and experiments, serve to validate the mathematical behavior described earlier.

**Figure 14:**
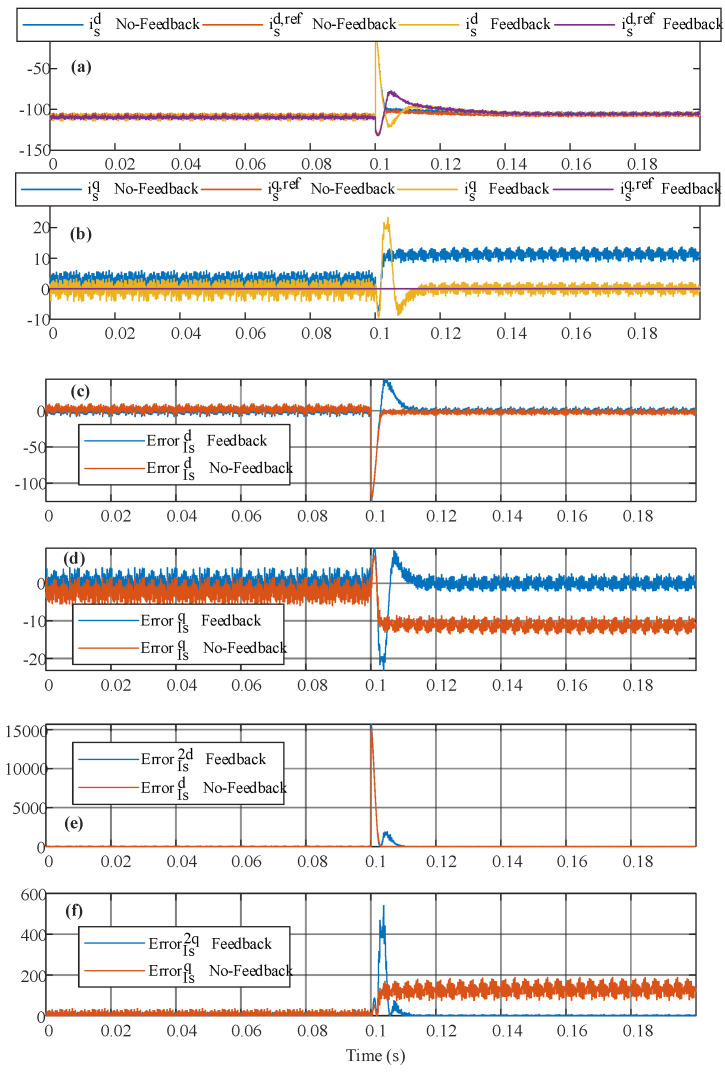
Comparison between the traditional MPC and the proposed method including stated feedback with integrator (**a**) direct current behave, (**b**) quadrature current behave, (**c**) direct current error, (**d**) quadrature current error, (**e**) square direct current error, and (**f**) square quadratic current error.

The initial results include a comparison between traditional MPC, which optimizes the current setpoint, and the proposed method with an integrator, which accounts for the past values of the variables. As shown in Figure 14a,b, the proposed method ensures zero steady-state error even with a 50% change in the inductance losses (resistor), while traditional MPC deviates significantly from the desired value. It is important to note that not only parameter changes can lead to steady-state error but also unmodeled nonlinearities, such as switch non-idealities, input filter frequency dependence, and minor sensing errors.

Figure 14 clearly shows that traditional MPC without feedback exhibits significant steady-state error, especially under parameter changes. The error relative to the reference is evident in Figure 14c,d, where the steady-state error is particularly pronounced. To quantify the improvement in the error reduction, the mean squared error is calculated for the direct and quadrature currents as follows:

(90) $$E_{d}^{2}=\frac{1}{N+1}\sum_{n=0}^{N}{e_{d}}^{2}\left(n\right),\;E_{q}^{2}=\frac{1}{N+1}\sum_{n=0}^{N}{e_{q}}^{2}\left(n\right)$$

Here, *e_d_*(*n*) and *e_q_*(*n*) represent the actual error for the direct and quadrature components relative to the reference, respectively, and *N* denotes the number of points considered. The quadratic error can be seen in Figure 14e,f, and the results are presented in Table 2, where the values are categorized by control type—whether the proposed feedback is included or not—and whether parameter deviations are present in the control. As shown in Table 2, the proposed control consistently results in a smaller mean squared error regardless of whether parameter deviations are present.

Thus, the reactive power, primarily associated with the quadrature current, due to the chosen synchronization, can effectively be controlled to follow the desired reactive power. This capability can be used to improve the power factor for other loads connected in parallel to the proposed topology. In other words, the proposed approach not only can inject active power but can also compensate for the power factor by ensuring the desired reactive power is maintained with zero steady-state error for predictive control.

### 6.1. Simulations

**Figure 15:**
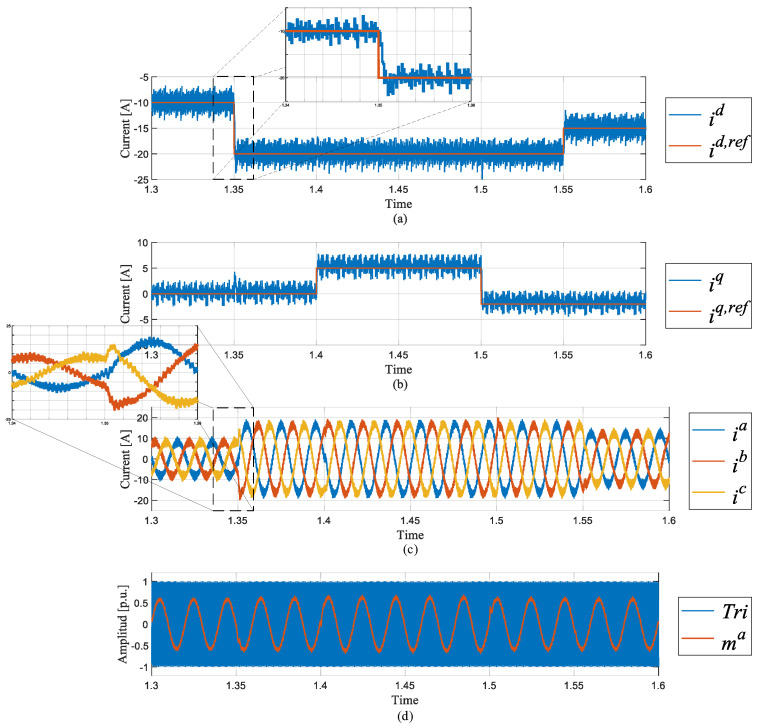
Simulation results: enhanced predictive control. (**a**) Direct current i^d^; (**b**) quadrature current i^q^, (**c**) three-phase grid current **i**^abc^, (**d**) modulator signal m^a^ vs. carrier tri.

In Figure 15, the enhanced predictive control loop is shown. Just as in Figure 9, the overshoot is visible in Figure 15a,b. The overall steady-state error is corrected with the modulation signal well under the overmodulation limit.

**Figure 16:**
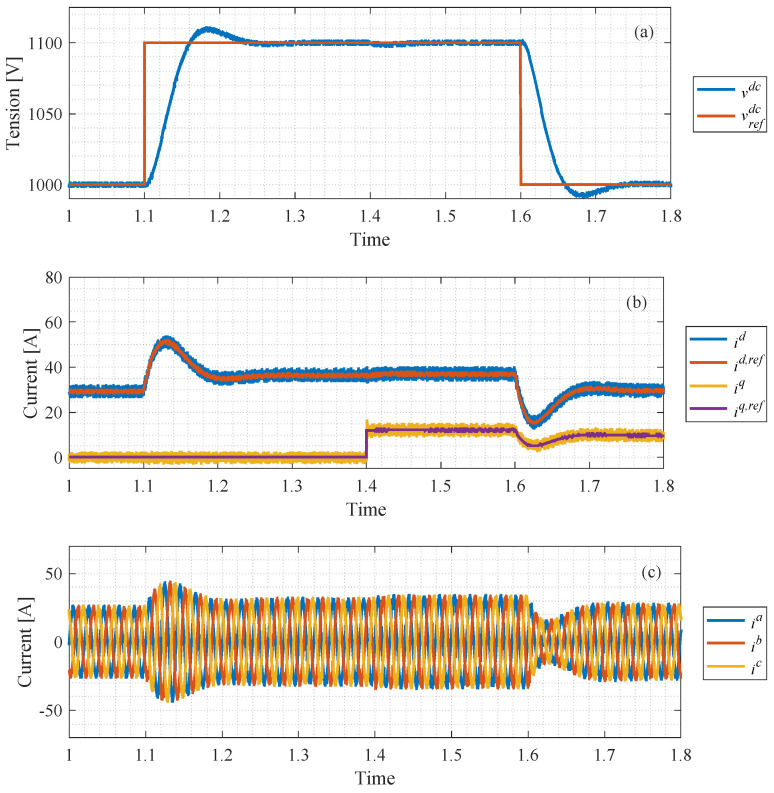
Simulation results: power control loop. (**a**) DC link capacitor voltage; (**b**) inner current control response; (**c**) three-phase grid current.

The dynamic response of the entire power/voltage control loop is shown in Figure 16. The step response of the DC voltage is presented in Figure 16a, and the output exhibits under 10% overshoot and a settling time of almost 1.6 ms, characteristics provided by the design. From the voltage control, the *dq*-current references are obtained for the inner enhanced predictive current control loop, and both the currents and the current references are shown in Figure 16b. Also, a step variation in the power factor, from unitary to 0.95 at *t* = 1.4 s, is presented in Figure 16b. It is clearly visible in the quadrature component of the *dq*-current, given (89), and its effect on the DC voltage is compensated for by the power control loop; thus, reactive power control is achievable. Lastly, the three-phase grid current is shown in Figure 16c.

**Figure 17:**
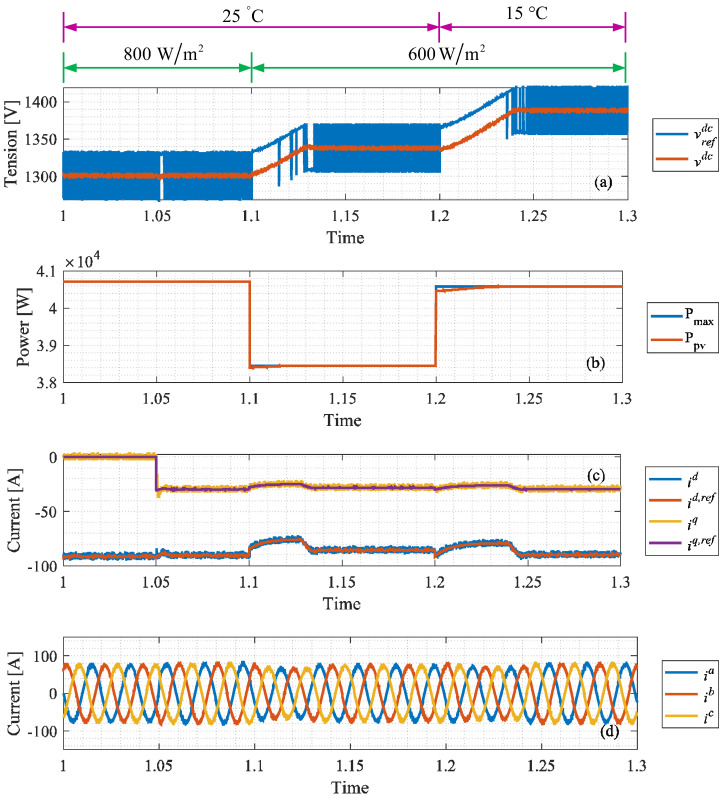
Simulation results: maximum power point tracking. (**a**) DC voltage; (**b**) PV array power; (**c**) dq-axis grid currents; (**d**) three-phase grid current.

In the context of solar applications, an MPPT algorithm is included in the control loop. Step changes for the irradiance and temperature are applied. Even though these types of changes are not normally encountered in real-life applications, these results provide a notion of the maximum power point tracking algorithm’s operation. In Figure 17, the temperature of the array varies from 25 °C to 15 °C at *t* = 1.2 s, and irradiance decreases from 800 W/m^2^ to 600 W/m^2^ at *t* = 1.1 s. As expected from the nature of the PV system, the power available rises at lower temperatures and decreases with lower irradiance, as shown in Figure 17b. The DC link voltage output is shown in Figure 17a, and references are obtained from the MPPT algorithm. Power factor variation from 1 to 0.95 is applied at *t* = 1.05 s, visible in the *q*-current shown in Figure 17c. The direct power is mostly related to the *d*-current, also shown in Figure 17c. The three-phase grid current is shown in Figure 17d.

### 6.2. Experimental Procedure

**Figure 18:**
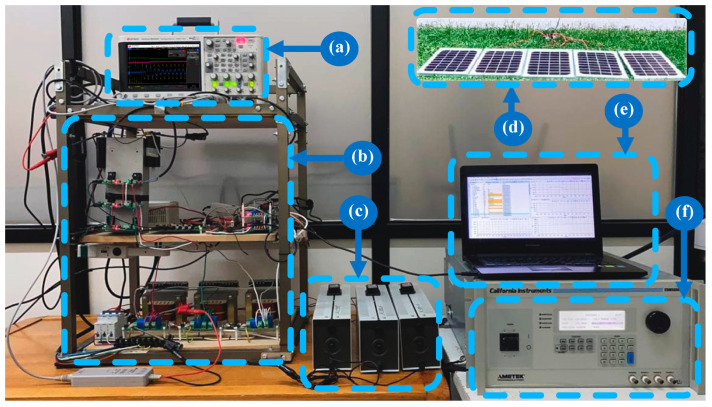
Experimental rig. (**a**) Oscilloscope; (**b**) voltage-source inverter–inductor–sensors–conditioning circuit–DSC; (**c**) resistive load; (**d**) PV array; (**e**) laptop for programming the DSC; (**f**) three-phase AC programming source.

To observe the real performance of the proposed control and topology, a prototype setup was constructed. The system was powered by a California Instruments programmable power source and fed by real solar panels installed in the laboratory’s front yard. Details of the setup can be seen in Figure 18.

**Figure 19:**
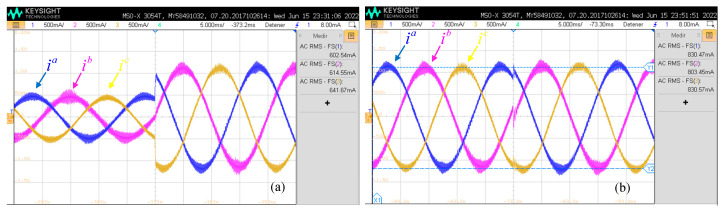
Experimental results: enhanced predictive control. (**a**) Direct current step response; (**b**) quadrature current step response.

In the one hand, Figure 19a shows the step response in the d component in the grid currents. Clearly, these changes have an effect on the amplitude of the three-phase grid current. On the other hand, step variations in the q-current change the phase of the grid currents, as shown in Figure 19b. The rise time of the response is close to 260 µs, which is close to three sampling periods. When compared to the ideal results in Figure 9a or Figure 9b, the experimental results show excellent performance.

**Figure 20:**
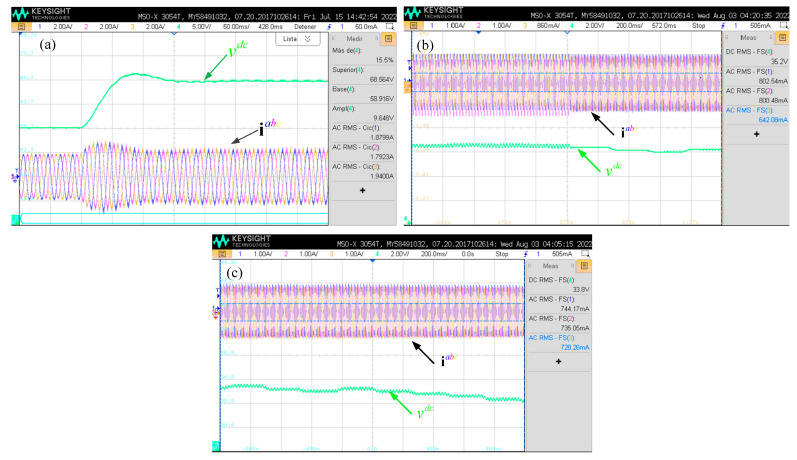
Experimental results: power control loop step response. (**a**) DC voltage response; (**b**) MPPT open-to-closed loop transition; (**c**) MPPT working.

The DC voltage control necessary for MPPT is shown in Figure 20a. The control loop, as presented in Figure 20a, was designed for 10% overshoot and an 8 ms settling time. When compared to the results expected from the simulation (Figure 16), it is noticeable that the experimental results in Figure 20a have 5% more overshoot, which may be due to the uncertain DC capacitor and resistance values.

In Figure 20b, the transition between the open and closed loops of the converter is shown. It is visible in the latter half that the shape of the currents improves, and the behavior of the MPPT is similar to the simulated results. Meanwhile, in Figure 20c, a closed-loop response is shown. The voltage tracking is clearly visible, as are the balanced three-phase currents.

## 7. Conclusions

Predictive control stands out as a leading method for controlling power converters due to its versatility, rapid response, easy implementation, and high performance. However, a notable drawback is its inherent lack of steady-state error correction, as traditional model predictive control optimizes instantaneous rather than average values. This paper addresses this limitation by proposing state feedback control combined with predictive control, preserving the advantages of predictive control while ensuring zero steady-state error. Furthermore, this study explores the robustness and sensitivity of the proposed approach, demonstrating its effectiveness through sensitivity response analysis and eigenvalue placement, which confirm the system’s stability even under significant parameter variations. The simulation and experimental results validate the designed controller’s ability to demonstrate satisfactory performance characteristics, as intended. This paper details the modeling of the power converter, emphasizing accurate equation tracking and replication of the proposed approach. Simulation and experimental validations further substantiate the effectiveness of the control strategy proposed. Additionally, the experimental setup includes a photovoltaic injection system with a power control mechanism regulating the DC voltage and power factor. The master loop employs a maximum power point tracking method based on traditional perturb and observe techniques, showcasing the robustness and performance of the proposed control strategy. Finally, the predictive controller proposed exhibits linear behavior, facilitating the application of linear control techniques such as optimal control to enhance its steady-state characteristics. However, future work should explore nonlinear variations to further advance the field.

## Figures and Tables

**Table 1:** Converter parameters.

Parameter	Value	
*R_f_* (line resistance)	1	Ω
*L_f_* (line inductance)	10	mH
*V_s_* (RMS grid voltage)	220	V
ω (angular frequency)	2π50	rad/s
*G_ac_* (converter gain)	0.5	p.u.
*Vdc* (DC voltage)	1000	V
*Ts* (sampling period)	100	µs

**Table 2:** Squared mean error.

Squared Mean Error	Type of Control	Parameter Deviations?	Values
$E_{d}^{2}$	No Feedback	Yes	8.0001
$E_{q}^{2}$	No Feedback	Yes	127.9266
$E_{d}^{2}$	Feedback	Yes	4.0722
$E_{q}^{2}$	Feedback	Yes	1.3007
$E_{d}^{2}$	No Feedback	No	17.4967
$E_{q}^{2}$	No Feedback	No	9.9013
$E_{d}^{2}$	Feedback	No	12.9180
$E_{q}^{2}$	Feedback	No	3.7939

## Data Availability

The data presented in this study are available on request from the corresponding author.
